# *Echinococcus multilocularis*: An Emerging Pathogen in Hungary and Central Eastern Europe?

**DOI:** 10.3201/eid0903.020320

**Published:** 2003-03

**Authors:** Tamás Sréter, Zoltán Széll, Zsuzsa Egyed, István Varga

**Affiliations:** * Central Veterinary Institute, Budapest, Hungary; † Szent István University, Budapest, Hungary

**Keywords:** *Echinococcus multilocularis*, red fox, Hungary, Central Eastern Europe, alveolar echinococcosis, emerging zoonosis, dispatch

## Abstract

*Echinococcus multilocularis*, the causative agent of human alveolar echinococcosis, is reported for the first time in Red Foxes (*Vulpes vulpes*) in Hungary. This parasite may be spreading eastward because the population of foxes has increased because of human interventions, and this spread may result in the emergence of alveolar echinococcosis in Central Eastern Europe.

*Echinococcus multilocularis* is the causative agent of alveolar echinococcosis in humans. The life cycle of this tapeworm is indirect and sylvatic; eggs shed by the definitive host, mainly the Red Fox (*Vulpes vulpes*) in Europe, develop to the metacestode stage in arvicolid rodents, which serve as intermediate hosts. In accidental cases, humans as aberrant intermediate hosts may also acquire *E. multilocularis* infection by egg ingestion. Although a rare disease in humans, alveolar echinococcosis is of considerable public health importance because it can be lethal in up to 100% of untreated patients (*1*). Treatment is still difficult, and therapy may cost $300,000 per patient (*1*).

The parasite has an extensive geographic distribution in the Northern Hemisphere, including parts of North America (Alaska, Canada, and some of the lower contiguous states of the United States), Asia (some of the newly independent states of the former Soviet Union, China, and Japan), and some European countries. Until the end of the 1980s, parasite-endemic areas in Europe were known to exist only in France, Switzerland, Germany, and Austria (*2*). In the 1990s and early 2000s, the infection rate of foxes increased drastically in some areas of France and Germany; several new endemic foci were detected in Switzerland, Germany, and Austria; and the parasite was reported from the surrounding countries, including the Netherlands, Belgium, Luxembourg, Poland, the Czech Republic, the Slovak Republic, and Italy (*1*,*3*,*4*). Here we report *E. multilocularis* infection from Red Foxes in the northern areas of Hungary and give a possible explanation for the spreading of the parasite from the west to the east.

Carcasses of Red Foxes sent to the Central Veterinary Institute, Budapest, from January to July 2002, in connection with the rabies immunization and control program, were included in this study. Carcasses were transported and stored in individual plastic bags at 4°C. The approximate delay between death and necropsy was 2 days. We examined the intestinal tracts by the sedimentation and counting technique as described (*5*). The whole sediment was examined in petri dishes under stereomicroscope at a magnification of X66, and worms were ascertained, counted, and subsequently washed and stored in 70% ethanol until DNA purification. Of 100 foxes (18 subadults and 82 adults) examined during the screening of the parasitologic status of the foxes in 15 counties in Hungary, 5 adults shot in April and May 2002 were found to be infected with 2, 3, 5, 6, and 254 mature worms of *Echinococcus*, respectively (Figure 1).

**Figure 1:**
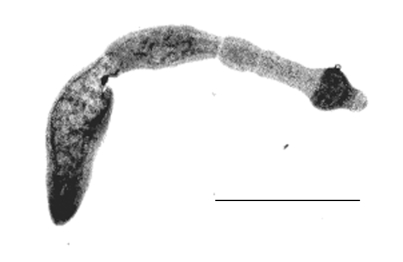
*Echinococcus* *multilocularis* isolated from a fox in Hungary. Scale bar: 0.5 mm.

All five foxes were shot in two northern Hungarian counties, Nógrád and Borsod-Abaúj-Zemplén, in the Nógrád Basin (Drégelypalánk, 48°02′ North, 19°04′ East, and Pusztaberki, 47°58′ North, 19°11′ East), in the Cserhát Mountains (Salgótarján, 48°03′ North, 19°47′ East and 48°01′ North, 19°45′ East) and in the Borsod Basin (Kelemér, 48°19′ North, 20°27′ East), near the Hungarian-Slovak border. The five places are in the Northern Mountain Range and at a distance of 60–120 km from the nearest known *E. multilocularis*–endemic region, the Muránska Planina Mountains (48°44′ North, 20′°02′ East) in Slovakia (*6*). These territories are 200–400 m above sea level and are mainly forested, nonagricultural, or extensive agricultural areas. Based on the most important morphometric parameters of *Echinococcus* adult stages (length of the worm: 1.3–2.5 mm; number of proglottids: 3–5; length of terminal proglottids: 0.5–1.1 mm; terminal proglottids in percentage of total worm length: 26–44; position of genital pore: anterior to middle; form of uterus: sacklike without lateral sacculations), the parasites were identified as *E. multilocularis* (*7*). Although the overall prevalence of *E. multilocularis* seems to be low in Hungary, in the two *E. multilocularis*–endemic counties, the prevalence was considerably higher (5 of 17 foxes were infected). These prevalence data are similar to those observed in the surrounding countries, Austria and Slovakia (*8*,*9*).

The taxonomic status of the isolates was also confirmed by a diagnostic polymerase chain reaction (PCR) assay. The five isolates were treated separately. Two different genes coding for U1 snRNA and the mitochondrial 12S rRNA genes have been used in diagnostic PCR for detecting *E. multilocularis* DNA (*10*–*12*); however, the species specificity of the PCR amplifying a fragment of the U1 snRNA gene could not be confirmed in a study (*12*). Thus, the nested PCR method described by Dinkel et al. (*11*) was used in our study. To exclude the possibility of contamination with specific DNA, a negative control was included and underwent the entire procedure starting with DNA extraction. DNA was purified as described (*13*). PCR reactions were performed by using a GeneAmp 2400 PCR system (PerkinElmer, Foster City, CA). The conditions used for PCR were identical to those described (*12*). PCR products were detected on ethidium bromide–stained 1.5% agarose gels by visualizing them with UV light. PCR products of the expected size (373 bp and 250 bp) were amplified in all cases (Figure 2), confirming the results of the morphologic comparisons, i.e., *E. multilocularis* was responsible for all infections.

**Figure 2:**
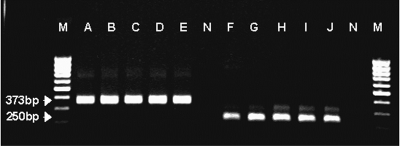
Nested polymerase chain reaction amplification of mitochondrial 12S rRNA gene from five Hungarian *Echinococcus multilocularis* isolates. Lanes A−E: amplification with outer primers; lanes F−J: amplification with inner primers; N, appropriate negative controls; M, molecular weight marker (100 bp).

According to some authors (*1*,*2*), researchers cannot confirm whether *E. multilocularis* is spreading from historically known *E. multilocularis*–endemic foci (eastern France, southern Germany, northern Switzerland, and western Austria) to new regions, or whether the Central European *E. multilocularis–*endemic area is connected with the *E. multilocularis*–endemic area in Asia, and the tiny worms previously escaped the attention of parasitologists. Our findings may suggest that the parasite’s range has recently expanded, rather than the first identification of formerly unknown *E. multilocularis*–endemic areas. The parasite was not identified previously in either Red Foxes or wild rodents in Hungary, despite the extensive studies conducted by Murai, Mészáros, Gubányi, and other parasitologists of the Natural History Museum, Budapest. Moreover, human cases have never been reported in Hungary. The photograph and the description of macroscopic lesions (two fist-sized, undulating cysts) in the only presumed report of alveolar echinococcosis written by two surgeons (*14*) clearly indicate that the case was indeed cystic echinococcosis.

The appearance of *E. multilocularis* in Hungary might be explained by changes in the size of the Red Fox population in central and Central Eastern Europe. From the 1970s, a continuous increase in the size of the Red Fox population was observed in Switzerland and Germany, probably as a consequence of the initiation of the antirabies vaccination programs (*2*). The larger population led to a continuous migration of young foxes from territories with high population density toward those with lower density, i.e., partly eastward. This migration might have resulted in the appearance of foxes infected with *E. multilocularis* and the establishment of small disease-endemic foci in Poland and the Czech Republic. After the political changes of 1990, considerable changes in land use were observed in the former communist countries because of the disintegration of large state farms. The probable consequences of these changes, the decrease of annual hunting index resulting from a decrease in the price of fox fur, and the initiation of antirabies vaccination of foxes in Central Eastern European countries (Poland, the Czech Republic, the Slovak Republic, and Hungary), caused a corresponding increase in the fox population size (*8*,*15*), and probably the coincidental increase of *E. multilocularis* population and prevalence and the expansion of *E. multilocularis–*endemic regions. A similar positive correlation between the population size of foxes and the prevalence of the parasite was also observed in Switzerland and Germany (*2*).

In the historically known *E. multilocularis–*endemic region, almost 400 patients are currently under continuous therapy, and the annual incidence of human alveolar echinococcosis has not varied markedly in the past few decades (*1*,*2*). In contrast with the stable epidemiologic situation in that region, the first 16 sufficiently documented and undoubtedly confirmed autochthonous human infections have been reported in Central Eastern European countries only from the late 1990s (*2*,*9*). Based on Central European annual incidence data (approximately 0.1–0.3/100,000 population) (*2*) and the similar overall prevalence of infection in foxes in Central and Central Eastern European countries (*2*,*9*), hundreds of cases would have been expected in the past few decades. The tiny worms may have escaped the attention of Central Eastern European parasitologists earlier. However, failing to recognize the characteristic and extensive lesions in humans in the past is unlikely.

Data from the Netherlands, Italy, Hokkaido Island and the surrounding islands of Japan, and North America provide clear evidence for the spreading and emergence of *E. multilocularis* infection (*2*,*4*,*12*). In the past, *E. multilocularis* has spread from the tundra zone of Northern Canada to the central regions of the continental United States (*7*) and from a small focus to the entire Hokkaido Island (*2*). Based on the above data, a similar spreading and emergence are likely being observed in Central Eastern European countries. As a result of their increasing population, foxes are inhabiting urban areas in several European countries, including Hungary (*1*,*15*). The appearance of foxes in a synanthropic environment may result in the infection of domesticated dogs and cats and may increase the risk for human infections in *E. multilocularis–*endemic areas. Thus, knowing that *E. multilocularis* is likely to continue to spread, one can predict that human alveolar echinococcosis will become an emerging infectious disease in Central Eastern European countries in a few years as has already occurred in some other European countries, Hokkaido Island of Japan, Canada, and the United States (*2*,*4*,*12*,*16*).
